# Metagenomic Analysis of Tick-Borne Viruses Associated With *Hyalomma asiaticum* From Different Hosts in the Surrounding Areas of Urumqi, China

**DOI:** 10.1155/tbed/9985595

**Published:** 2025-11-10

**Authors:** Junxia Jin, Xiaoshan Chao, Zhongzheng Zhu, Beibei Zhang, Yujiao Fu, Juan Xu, Shuying Ma, Tianyi Chen, Ying Wang, Juntao Ding

**Affiliations:** ^1^College of Life Science and Technology, Xinjiang University, Urumqi, Xinjiang 830017, China; ^2^Xinjiang Key Laboratory of Biological Resources and Genetic Engineering, Urumqi, Xinjiang 830017, China; ^3^College of Textiles and Clothing, Xinjiang University, Urumqi, Xinjiang 830017, China

**Keywords:** *Hyalomma asiaticum*, phylogenetic analysis, tick-borne virus, Urumqi, viral metagenomics

## Abstract

Tick-borne viruses (TBVs) represent a serious risk to global public and animal health. Despite the remarkable species diversity of ticks documented in Urumqi and its adjacent regions of China, scientific investigations into TBVs in this ecologically significant area have been strikingly scarce. In this study, we conducted metagenomic sequencing on 752 *Hyalomma asiaticum* (*H. asiaticum*), including questing ticks from Wujiaqu and blood-feeding ticks collected from sheep and horses in the Changji area. A total of 11 different RNA viruses were detected, belonging to six viral families and some unclassified families, with double-stranded RNA viruses being the most prevalent (49.1%), including Totiviridae and Sedoreoviridae. Single-stranded RNA viruses accounted for 11.9% of the virome, encompassing Chuviridae, Flaviviridae, Rhabdoviridae, and Phenuiviridae. Notably, 39.0% of the viral sequences remained unclassified, highlighting a substantial reservoir of uncharacterized viral diversity. Genomic and phylogenetic characterizations were performed on six highly abundant viruses, including Bole tick virus 1, Mivirus boleense, Bole tick virus 4, Lonestar tick totivirus, Hubei toti-like virus 24, and a novel strain of Hulunbuir Totiv tick virus 1. However, their zoonotic potential requires further investigation. By integrating cytochrome c oxidase subunit Ⅰ (*COI*) gene-based tick species identification with viral metagenomics, this study provided a comprehensive assessment of tick species and TBVs diversity in Urumqi and its surrounding areas, China. These results provide new insights into both the genetic diversity of tick-borne RNA viruses and their phylogenetic connections, while also expanding knowledge about the geographical distribution patterns of these pathogens.

## 1. Introduction

Ticks represent a specialized group of hematophagous ectoparasites capable of transmitting the most diverse array of pathogenic microorganisms to vertebrate hosts [1]. With the exception of limited cases, all three major tick families (Ixodidae, Argasidae, and Nuttalliellidae) exhibit obligatory hematophagy throughout their life cycles. Ticks have long been established as significant vectors in both medical and veterinary disease transmission systems. They are the earliest recognized arthropod vectors of pathogenic agents. The late 19th century witnessed Smith and Kilbourne's groundbreaking identification of *Rhipicephalus* ticks as the vectors of bovine fever. Since then, the study of ticks' ability to acquire, maintain, and transmit pathogens has become an important and fruitful field of scientific research [2, 3].

Xinjiang, a crucial border region in northwestern China, has an economy predominantly based on agriculture and animal husbandry. This production paradigm results in frequent human exposure to arthropod vectors such as ticks and mosquitoes, substantially elevating the risk of zoonotic transmission of tick-borne pathogens [4, 5]. As early as 1965, an outbreak of Xinjiang hemorrhagic fever (XHF)—characterized by high fever, hemorrhage, and renal impairment—occurred in Bachu County, Xinjiang. A subsequent outbreak in the same region in 1997 resulted in 26 reported cases, including four fatalities, within a 45-day period [6]. In recent years, advances in viral detection technologies have enabled the identification of a series of novel viruses in ticks from Xinjiang, such as Tacheng tick virus (TcTV) types 1–2 [7]. Notably, a novel virus, Guertu virus (GTV), was discovered in 2016 in *Dermacentor nuttalli* ticks collected from Guertu County, Xinjiang. Phylogenetic analysis revealed that GTV belongs to the genus Bandavirus, closely related to SFTSV and HRTV [8]. Serological surveys further demonstrated that 92 out of 465 local resident serum samples (19.8%) tested positive for GTV antibodies, confirming the virus's capacity to infect humans. These findings collectively highlight the potential threat posed by tick-borne viruses (TBVs) to human health in the Xinjiang region.

The advent of next-generation sequencing (NGS), a high-throughput and efficient method, has significantly propelled the research on tick-borne virology and updated our understanding of the virome [9]. Since its first application on ticks in 2011 to study the diversity of bacterial composition, NGS has been increasingly employed in the discovery of TBVs over the past 5 years [10]. Several novel viruses have been identified worldwide, spanning continents such as the Americas, Africa, Asia, and Australia [11]. For instance, NGS-based analyses by Ni et al. [12] have detected hundreds of novel tick-borne RNA viruses, substantially enhancing our comprehension of the tick virome. However, there are still obvious limitations in the existing research, the differences in viruses carried by ticks from different host sources and their relationship with environmental factors still need to be further explored, and so far only limited ecological data of TBVs are available, and there is a lack of systematic survey data in important ecological areas in Xinjiang, China.

In this study, an advanced RNA virome analysis technique, metatranscriptomics (MTT), was used to characterize the RNA virome of ticks collected in Urumqi and surrounding areas, China. Samples included questing ticks from the Wujiaqu area and blood-feeding ticks obtained from sheep and horses in the Changji area. The objectives of our study were: (1) to elucidate the composition of the viral community, (2) to compare differences in viral carriage between populations, and (3) to perform genomic characterization and phylogenetic analyses of the identified TBVs. This study aims to reveal the diversity patterns of TBVs in Xinjiang, thereby providing important data for evidence-based prevention and control strategies for tick-borne and related viral infections.

## 2. Materials and Methods

### 2.1. Collection and Species Identification of Tick Samples

**Figure 1:**
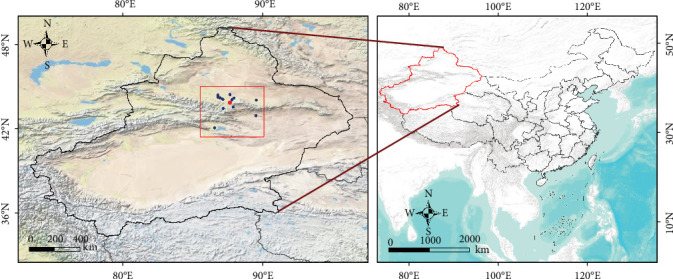
A map of the study area.

Field investigations were conducted in Urumqi and its surrounding areas from April to July in 2024, during which questing ticks and blood-feeding ticks were collected (Figure 1). The morphology of the ticks was observed using a stereomicroscope, and a preliminary identification of tick species was carried out by referring to “Economic Entomology of China, Volume 39, Tick and Mite Subclass, Hard Tick Family” for comparison [13]. To ensure the accuracy of species identification and avoid missing potential tick species, genomic DNA was extracted from individual tick using the QIAamp DNA Mini Kit (QIAGEN, Germany), followed by PCR amplification of the cytochrome c oxidase subunit Ⅰ (*COI*) gene with universal primers (Supporting Information Table S1) [14]. PCR products were electrophoresed on 1% agarose gels, and target-sized amplicons were purified for Sanger sequencing. The sequences were analyzed using blastn and used to construct phylogenetic trees.

### 2.2. Sample Preparation and RNA Extraction

To investigate the impact of different hosts on virus carriage by ticks, 15 ticks (all *Hyalomma asiaticum* [*H. asiaticum]*) were randomly selected and grouped strictly by host source for library construction, resulting in a total of three transcriptome libraries. The three libraries were labeled as F = questing ticks, H = blood-feeding ticks on horse and S = blood-feeding ticks on sheep. In order to improve the sensitivity of viral RNA detection, we used a virus enrichment strategy before extracting RNA. First, the tick samples stored at −80°C were thawed and homogenized with 100 μL PBS. Then, the impurities were removed by liquid nitrogen freeze–thaw and centrifugal filtration (0.45–0.22 μm filter membrane). The virus was enriched by sucrose density gradient ultracentrifugation (160,000 *g*, 2 h), and the precipitate was preserved at −80°C after enzymatic hydrolysis. After obtaining a relatively pure TBV-like particle sample, RNA extraction was conducted using the TaKaRa MiniBEST Viral RNA Extraction Kit Ver.5.0 (Takara Bio, Japan). Finally, 1 µL sample was taken and quantified using the Qubit4.0 (Thermo Fisher Scientific, USA). RNA extraction products were stored in an −80°C refrigerator to maintain stability for future experimental use.

### 2.3. Library Preparation

The ALFA-SEQ Directional RNA Library Prep Kit (Vazyme Biotech, China) was used for the qualified samples, and the library was completed according to the accompanying instructions. The quality of the library was evaluated by Qubit4.0 (Thermo Fisher Scientific, USA). Finally, PE150 sequencing of the constructed amplicon library was performed using the Illumina platform.

### 2.4. Quality Control, Adapter Removal, and De Novo Assembly

In view of the fact that the original data obtained from sequencing contains a certain proportion of low-quality reads, that is, containing spliced and repeated sequences, these reads will affect the subsequent analysis. In order to ensure the accuracy and reliability of the subsequent analysis results, we first use Trimmomatic (v0.39) [15] to preprocess the original sequencing data to obtain valid data for subsequent analysis. Subsequently, the BWA (v0.7.17) [16] software is employed to remove the contaminations of host sequences and ribosomal sequences. Thereafter, MEGAHIT (v1.1.3) [17] is adopted for de novo assembly. Meanwhile, the utilization rate of reads is calculated by the Sourmash (v4.8.4) software. Ultimately, the contigs of all samples are clustered to obtain unique contigs (commands and parameters: Supporting Information Table S2).

### 2.5. Virus Sequence Identification

To characterize RNA viruses in the tick virome while minimizing host-derived false positives, we implemented a rigorous bioinformatics pipeline. The known viruses were identified using blastn against the Viral-NT database with stringent thresholds (*e* ≤ 1 × 10^−5^, alignment length ≥ 500 bp). Putative novel viruses were predicted through consensus analysis using DeepVirFinder (v1.0), VirSorter2 (v2.2.4), and GeNomad (v1.8.0), followed by NT database validation (*e* ≤ 1 × 10^−8^). Remaining sequences were analyzed against the NR database using diamond (*e* ≤ 1 × 10^−5^) before final taxonomic classification via the NCBI system [18] (Commands and parameters: Supporting Information Table S2).

### 2.6. Virus Abundance Statistics and Intergroup Difference Analysis

Salmon (v0.13.1) [19] was used to align clean reads to each contig that has been identified as a virus, and the transcription of each virus contig per 1000 bases per million mapped transcript (TPM) was calculated. Prodigal (v2.6.3) was used to predict the open reading frames (ORFs) in the viral genome. The *G*-test test method of STAMP (v2.1.3) [20] software was used to analyze and visualize the differences in the composition of TBVs in different hosts and infer their abundance changes (commands and parameters: Supporting Information Table S2).

### 2.7. Virus Genome Assembly and Genetic Evolution Analysis

Based on the results of virus contigs annotation, the high similarity reference genome was obtained from NCBI, and BWA (v0.7.17) was used for sequence alignment [15]. The whole genome of the virus was assembled using Geneious11.0, and the genome was visualized using Proksee [21]. The virus genome was temporarily named according to the geographical characteristics of the sample area. The highly conserved RdRp sequence was selected to construct the phylogeny at the family level, and sequence alignment was performed using ClustalW and default parameter settings. Phylogenetic reconstruction was performed using maximum likelihood algorithms implemented in MEGA6 software package (https://megasoftware.net), and the phylogenetic tree was beautified by tvBOT [22].

### 2.8. Molecular Detection of TBVs by RT-PCR

Based on the metagenomic sequencing data, the present study targeted the conserved regions of TBVs, such as the RdRp gene. Specific primers were designed with Primer Premier 5 software to generate amplicons 200–1000 bp in length and were synthesized by Xinjiang Youkang Biotechnology Co., Ltd. (Table 1). Viral RNA isolation was performed with the TaKaRa MiniBEST Viral RNA Extraction Kit (Takara Bio, Japan). Subsequent genomic DNA elimination and cDNA synthesis were achieved using the PrimeScript RT Reagent Kit with integrated gDNA Eraser (Takara Bio, Japan). Using the cDNA extracted from each sample as template, viral genes were amplified by RT-PCR with the primers listed in Table 1. PCR products were examined by 1% agarose gel electrophoresis, and positive bands of the expected size were selected for Sanger sequencing. The PCR reaction mixtures and cycling conditions are detailed in Tables 2 and 3, respectively.

## 3. Results

### 3.1. Species Identification of Ticks

A total of 236 questing ticks were collected at Wujiaqu City, 222 blood-feeding ticks were collected on the surface of sheep in Changji City, and 294 blood-feeding ticks were collected on the surface of horses. Referring to the search table in “Economic Entomology of China, Volume 39, Tick and Mite Subclass, Hard Tick Family,” the morphological identification of the collected ticks was carried out by stereomicroscope. The preliminary identification results showed that the collected ticks were *H. asiaticum* (Supporting Information Figure S1). In order to further confirm the tick species, genomic DNA of ticks was subject to *COI* gene PCR amplification and sequencing. After comparison, it was found that its identity with the *COI* gene of *H. asiaticum* isolate GY20 (MN865123.1) reached 99.7%, thus confirming that the collected tick sample was *H. asiaticum*, which was the dominant tick species in Xinjiang. This finding provides an important species basis for further study of tick-borne diseases in this area (Supporting Information Figure S2).

### 3.2. High-Throughput Sequencing and Assembly

We performed NGS of both blood-feeding and questing ticks samples on the Illumina NovaSeq 6000 platform, successfully constructing three transcriptome libraries that generated 45.3 Gb of raw sequencing data. After removing the low-quality and host sequences in the original reads, a total of 2,262,262 valid reads were obtained for subsequent analysis. Among them, bioinformatic analysis revealed 34,931 sequencing reads exhibiting significant alignment to known viral protein sequences in the GenBank NR database. The number of virus-related reads per library varied from 97 to 20,962, with viral matches detected in all libraries. Postquality control and removal of ribosomal and host contaminants, de novo assembly of each library was performed using MEGAHIT (v1.1.3). CD-HIT (v4.7) clustering of the assembled contigs yielded 255 unique sequences, with an average N50 of 3091 bp (Table 4).

### 3.3. Virus Sequence Analysis

**Figure 2:**
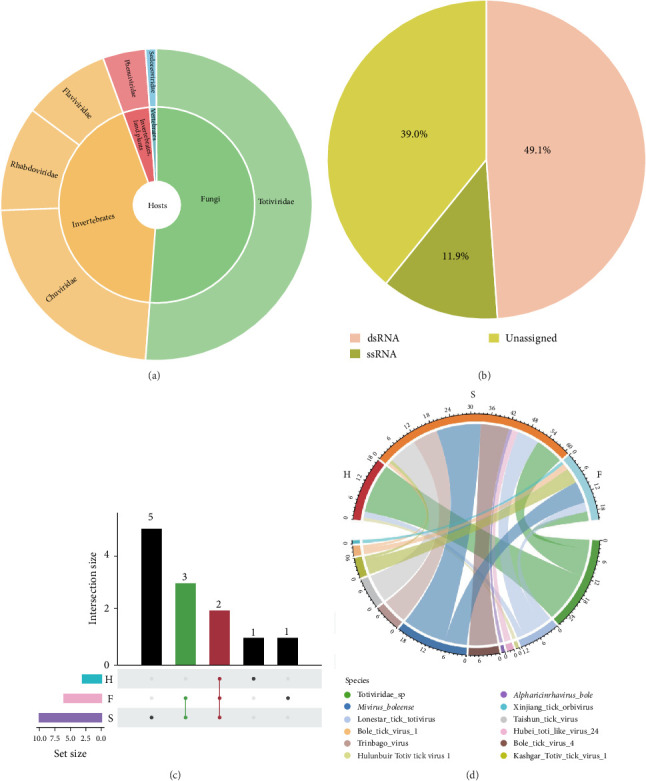
Macrovirus sequencing species classification annotation. (A) Classification of all host-derived viral sequences, (B) viral sequences of different nucleic acid types, (C) upset plot showing species shared and endemic to each sample, and (D) the number distribution map of RNA virus contigs at the species level was detected in the TBVs library. F, questing ticks; H, blood-feeding ticks on horse; S, blood-feeding ticks on sheep.

A total of 34 confirmed viral contigs were identified by comparison with the GenBank nonredundant database, and 215 suspected viral contigs were screened using DeepVirFinder (v1.0), VirSorter2 (v2.2.4), and GeNomad (v1.8.0) software (Supporting Information Table S4, Figure S3). A total of 249 virus contigs were annotated to six virus families and some unclassified viruses, among which Totiviridae, Chuviridae, Rhabdovirida, and Flaviviridae were the most widely distributed (Supporting Information Figure S4). The host-derived prediction results showed that the viral community mainly included vertebrates, invertebrates, plants, and fungal-derived viruses (Figure 2A). It is worth noting that 49.1% of the viruses are dsRNA viruses, 11.9% are ssRNA viruses, and 39.0% are unclassified viruses, indicating that the tick virus group has a specific preference for dsRNA (Figure 2B). These results further reveal the high diversity of TBVs.

According to the results of taxonomic comparison, 11 viruses and some unclassified viruses were identified from the existing database, of which two viruses coexisted in the three groups of libraries (Figure 2C). After analyzing each sample, it was found that S sample showed the highest virus diversity. A total of eight viruses were detected in this sample, accounting for 72.7% of the total number of annotated viruses. The viruses with the highest proportion were Mivirus boleense and Taishun tick virus. Five viruses were annotated in the F sample. It is worth noting that Mivirus boleense also had a high abundance in this sample. Different from the above two samples, the virus diversity of H sample was the lowest, which contained only 18.2% of the total number of annotated viruses, and in this sample, most of the viruses were annotated as Totiviridae sp., accounting for 84.0% of the sample (Figure 2D). Numerous studies have demonstrated that ticks are unable to transmit viruses within the population without vertebrate hosts as intermediate media [23], suggesting that the RNA viruses they carry may be stable members of the microbiome, and their community differences may be related to the host environment and vertical transmission mechanisms.

### 3.4. Virus Abundance Statistics and Ecotype-Associated Patterns

**Figure 3:**
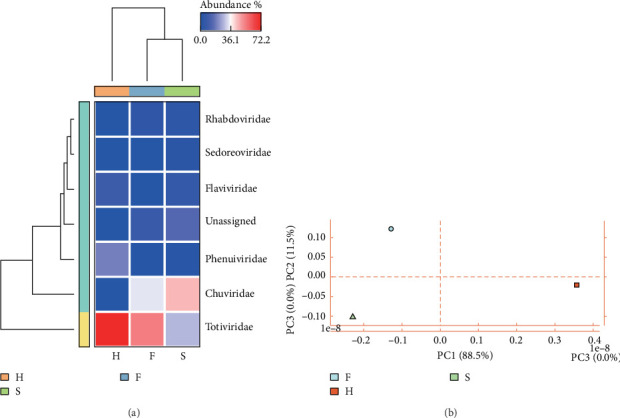
Viral abundance and intergroup differences analysis. (A) The heatmap of relative abundance for the remaining viral families and (B) PCA of viral community composition. Samples are color- and shape-coded by tick ecotype (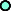: F, questing ticks; 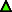: S, blood-feeding ticks on sheep; 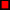: H, blood-feeding ticks on horse). Principal components explain 88.5% (PC1) and 11.5% (PC2) of total variance.

Through quantitative analysis by salmon software, Totiviridae was the most abundant in H and F samples, while Chuviridae was dominant in S sample (Figure 3A). PCA indicated that the F, H, and S samples showed a tendency to separate in the principal component space, with the most obvious distinction between F and H samples, and samples within each group (e.g., S sample) showed a certain degree of aggregation, suggesting a potential separation trend in virome composition among different tick ecotypes (Figure 3B). It should be noted that this PCA result is a descriptive presentation of the overall distribution pattern of the virome, and due to the limitation of the sample pooling strategy (only one library per group without biological replicates), statistical testing of intergroup differences cannot be performed. These observations provide preliminary insights into the potential association between tick ecotypes and virome composition, which may contribute to understanding the host specificity of TBVs transmission.

### 3.5. Genome Characteristics and Phylogenetic Analysis of Tick-Associated RNA Viruses

The complete genome sequences or ORF of the five viruses were obtained from three samples, and the virus genomes were named according to the geographical characteristics of the collected areas. The phylogenetic analysis at the family level was constructed to characterize the tick-related viruses (Table 5).

#### 3.5.1. Phenuiviridae

**Figure 4:**
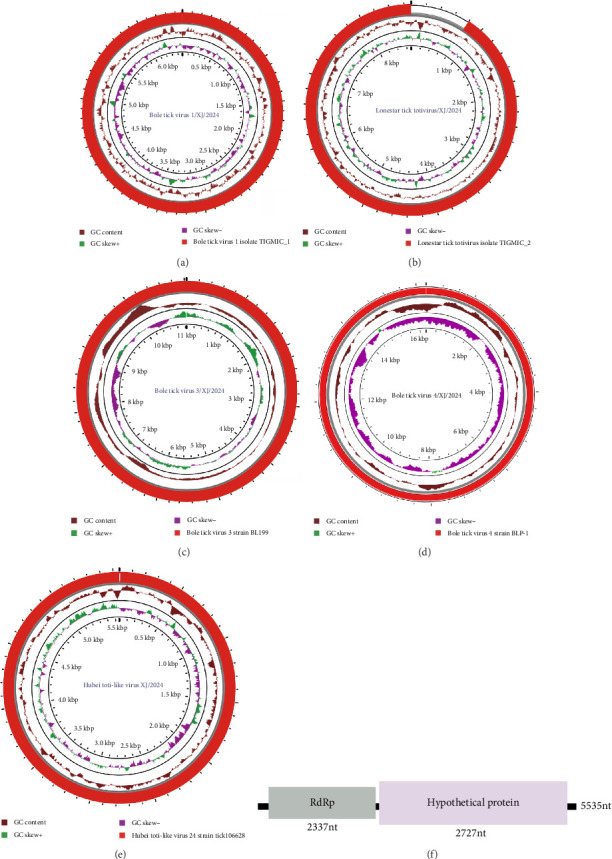
Schematic diagram of the whole genome structure of tick-borne virus. The complete genome structures of Bole Tick Virus 1/XJ/2024 (A), Lonestar tick totivirus (B), Bole Tick Virus 3/XJ/2024 (C), Bole tick virus 4/XJ/2024 (D), Hubei toti-like virus 24/XJ/2024 (E), and Hulunbuir tick virus 1/XJ/2024 (F) were shown in the circle plots, respectively.

**Figure 5:**
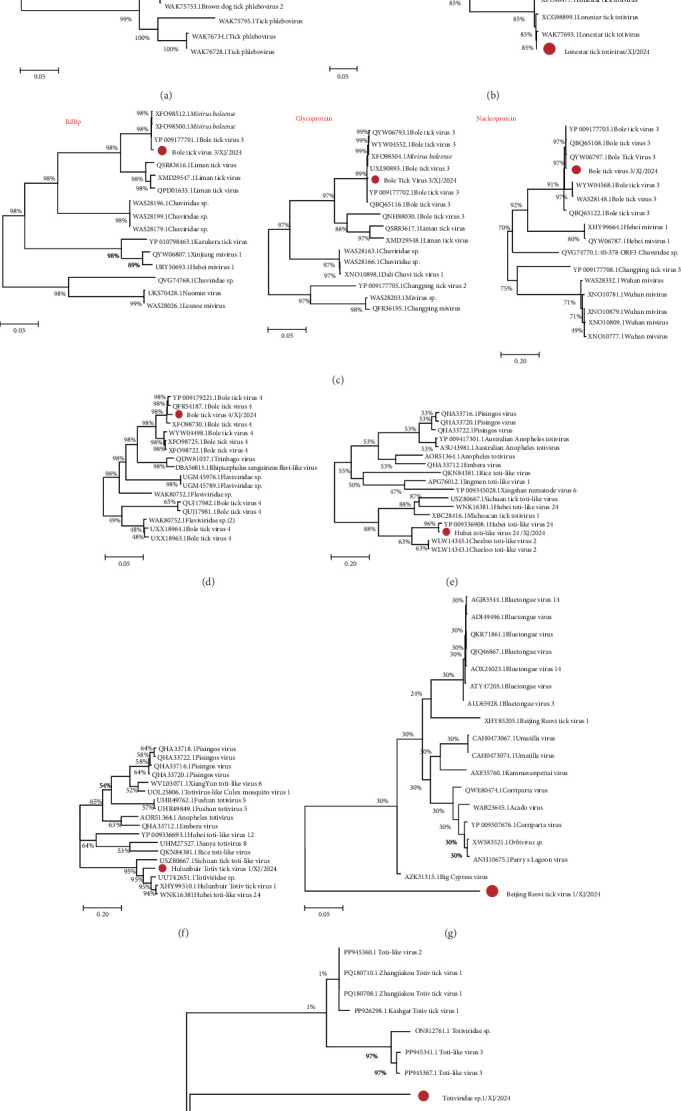
Phylogenetic relationships of the viruses identified from ticks. Phylogenetic trees were reconstructed using the complete amino acid sequences of the conserved viral proteins from the following strains: Bole tick virus 1/XJ/2024 (A), Lonestar tick totivirus (B), Bole tick virus 3/XJ/2024 (C), Bole tick virus 4/XJ/2024 (D), Hubei toti-like virus 24/XJ/2024 (E), Hulunbuir tick virus 1/XJ/2024 (F), Beijing Reovi tick virus 1/XJ/2024 (G), and Totiviridae sp./XJ/2024 (H).

Bole Tick Virus 1/XJ/2024 exhibits a simplified genomic structure, encoding only RdRp, with no other nucleoprotein-encoding regions detected. In this study, a complete RdRp virus sequence was obtained, with a total length of 6475 bp and a GC content of 45.9% (Figure 4A). It shared the highest homology with Bole Tick Virus 1 isolate TIGMIC_1 (WAK75658.1) previously found in Xinjiang, China, and clustered into the same cluster on the phylogenetic tree (Figure 5A).

#### 3.5.2. Totiviridae

Lonestar tick totivirus is a virus that was recently discovered in the American dog tick. Our study obtained a complete viral sequence of Lonestar tick totivirus/XJ/2024, which is 8695 base pairs long with a GC content of 57.1%, and two ORF, encoding capsid protein (CP) and RdRp, respectively (Figure 4B). Phylogenetic analysis revealed 96.8% and 97.2% amino acid identity for CP and RdRp, respectively, with reference strain TIGMIC_2 (WAK77694.1), confirming its taxonomic position within Totiviridae (Figure 5B).

#### 3.5.3. Chuviridae

Bole tick virus 3/XJ/2024 is a single-stranded negative-sense RNA virus with a genome of 11,038 bp and a GC content of 49.0%. It contains three ORF and encodes RdRp, GP, and NP. The genome structure (L-G-N arrangement) was consistent with the characteristics of Chuviridae (Figure 4C). The new strain had high homology with the reference strain Bole tick virus 3 strain BL199 (YP_009177701.1) in the amino acid sequences of RdRp, GP, and NP proteins (99.3%, 99.5%, and 99.4%, respectively). Phylogenetic analysis using RdRp, GP, and NP aa sequences showed that all Bole tick virus 3 strains formed an evolutionary branch (Figure 5C).

### 3.6. Unclassified Viruses

In recent years, the extensive use of NGS has led to the identification of numerous novel virus-related sequences in various tick species. In this study, we subjected family-unclassified viruses to integrated genomic and phylogenetic analyses. We recovered three near-complete viral genomes whose species-level identities were unambiguously resolved, together with two contigs that either lack known species counterparts or share <50% amino-acid identity with the closest recognized taxa. Bole tick virus 4/XJ/2024 is a 16,234 bp flavivirus-like virus with a single polyprotein ORF (Figure 4D). The virus clustered with the Chinese strain Bole tick virus 4 strain BLP-1 (YP_009179221.1), showing 98.3% amino acid identity (Figure 5D). Hubei toti-like virus 24/XJ/2024 (5797 bp), containing two putative protein genes (Figure 4E), formed a close phylogenetic cluster with the Chinese reference strain Hubei toti-like virus 24 strain tick 106628 (YP_009336907.1), demonstrating strong evolutionary conservation (Figure 5E). We obtained partial sequences (5535 bp, GC 54.7%) encoding an RdRp and a hypothetical protein, designated as Hulunbuir tick virus 1/XJ/2024 (Figure 4F). Phylogenetic analysis based on RdRp sequences revealed 80.9% amino acid identity to the known Hulunbuir tick virus 1 for the protein (Figure 5F). Furthermore, two viral contigs with less than 50% sequence identity to known species exhibited distinct evolutionary characteristics. Specifically, Beijing Reovi tick virus 1/XJ/2024 shared only 49.8% sequence identity with the homologous virus Beijing Reovi tick virus 1 (XHY85205.1) previously reported in China and formed a separate clade in the phylogenetic tree, indicating significant genetic divergence from known homologous viruses (Figure 5G). In contrast, Totiviridae sp./XJ/2024 clustered into an independent clade on the phylogenetic tree, belonging to a different evolutionary lineage from related viruses such as Toti-like virus 3 with significantly lower sequence identity (Figure 5H). These findings further highlight the genetic diversity and potential novelty of unclassified viruses in the study area.

### 3.7. Molecular Detection of TBVs by RT-PCR

**Figure 6:**
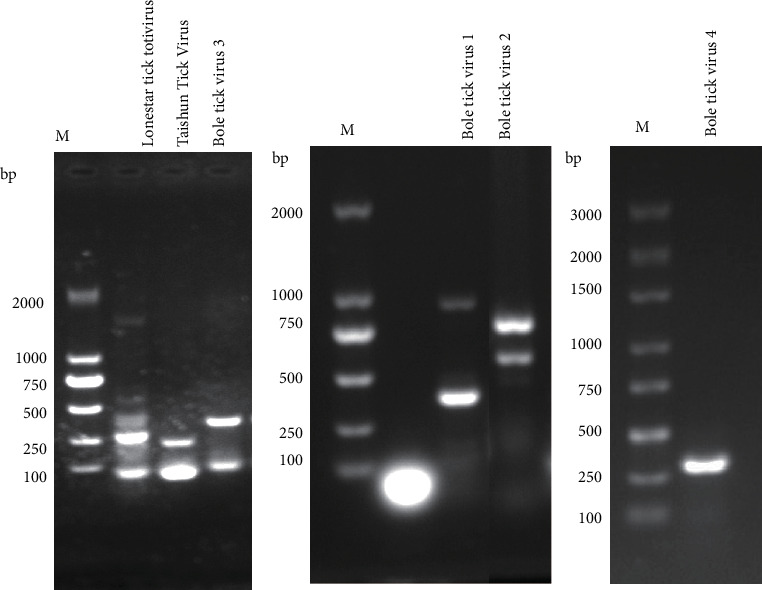
The results of virus RT-PCR amplification.

The metagenomic results revealed that 11 viruses were annotated in both parasitic and free-living ticks, which were subsequently confirmed by PCR. As shown in Figure 6, the PCR amplification results demonstrated the successful amplification of target bands for Bole tick virus 1 (952 bp), Bole tick virus 2 (763 bp), Bole tick virus 3 (344 bp), Bole tick virus 4 (299 bp), Lonestar tick totivirus (264 bp), and Taishun tick virus (217 bp). Multiple bands observed in some lanes may result from nonspecific priming or secondary structures in viral RNA.

## 4. Discussion

As hematophagous arthropod vectors of global significance, ticks impose dual burdens on livestock populations through both direct blood-feeding consequences and transmission of diverse viral, bacterial, and protozoan pathogens to vertebrate hosts [24, 25]. Urumqi, the regional capital of Xinjiang Uygur Autonomous Region, serves as the core administrative and socioeconomic nucleus while functioning as a critical nexus for transCentral Asian connectivity. Its unique geographical location and prosperous commercial activities have brought about a highly dense population flow, while the vast surrounding agricultural and pastoral areas provide an ideal habitat for a variety of ticks. More than 10 major tick-related diseases have been identified in the surrounding areas of Urumqi, China [26, 27]. The area is rich in tick species, among which *Ixodes persulcatus*, *Dermacentor nuttalli*, *Dermacentor marginalis*, *Dermacentor silvarum*, *Dermacentor argenteus*, *H. asiaticum*, *Hyalomma vestiges*, and other dominant tick species in this area have been confirmed [28]. This study focuses more on the more abundant counties and cities in Xinjiang, such as Xinjiang population, livestock, and insects. We choose different hosts to obtain tick information and investigate the intensity of its distribution. However, studies have found that some alien or invasive species will undergo genetic mutations or evolution due to differences in the climate, geographical habitats, and other living environments of the new environment, which can easily lead to the phenomenon of tick species identification errors [4]. Therefore, the application of a fast and efficient DNA barcoding marker is essential for tick species identification. In this study, adult ticks collected by morphological identification were investigated and molecular identification was performed. In this study, the adult ticks collected were identified by morphological and molecular methods and the identity of *H. asiaticum* was confirmed.

Urumqi is one of China's significant livestock production bases, with sheep and horse farming playing a crucial role in the local animal husbandry industry. As competent vectors of viral pathogens, ticks facilitate disease transmission among domesticated ruminants and equids, resulting in considerable financial burdens for animal husbandry operations. Notably, certain TBVs circulating in Xinjiang exhibit zoonotic potential, with Crimean-Congo hemorrhagic fever virus (CCHFV) representing a critical example. This WHO-designated priority pathogen is primarily transmitted to livestock (e.g., sheep and horses) by *H. asiaticum*, with human infections occurring through contact with infected animal tissues or direct tick bites, resulting in case fatality rates approaching 30% in human cases [29, 30]. In this study, we carried out a survey of TBVs in *H. asiaticum* from different hosts. Although tick samples from certain key hosts (such as camels and rodents) were not collected in this study, the comprehensive analysis of ticks parasitizing on hosts like horses and sheep, combined with the investigation of questing ticks, demonstrated that ticks parasitizing on different hosts carry a rich diversity of TBVs. Specifically, 11 distinct viral species were identified in this study, covering at least six viral families, namely Chuviridae, Flaviviridae, Sedoreoviridae, Phenuiviridae, Rhabdoviridae, and Totiviridae. This is consistent with the high diversity of RNA viruses in ticks observed in previous studies. Moreover, among the viruses detected in this study, none are currently known to cause symptomatic diseases in humans or animals. However, they might potentially influence the ability of ticks to harbor and transmit pathogens. Consequently, it is necessary to conduct larger-scale and more systematic studies on ticks to uncover those viruses that could potentially lead to diseases in vertebrate animals.

The complex and diverse ecological environment in Xinjiang provides ideal conditions for the spread of TBVs [31]. The cograzing of diverse livestock species (cattle, sheep, horses, and camels), combined with abundant small mammals in densely vegetated areas, significantly enhanced tick-host exposure opportunities while reducing reliance on any single host species [4, 32]. This may be an important ecological basis for the diversity of local tick-borne diseases. It was found that host species significantly affected the composition of tick virome: the viral diversity of blood-feeding ticks on sheep was higher than that of free ticks, while that of blood-feeding ticks on horse showed the opposite trend. This phenomenon is different from the classical theory of 'blood-sucking virus' [33], suggesting that horse blood may contain viral inhibitors such as antimicrobial peptides. Subsequent studies need to combine the life history characteristics of ticks and host habitat preferences to deeply analyze the complex relationship between host body size and viral load.

This study identified and characterized a diverse array of TBVs from various families including Phenuiviridae, Totiviridae, Chuviridae, and unclassified viruses. Our analysis revealed the presence of six distinct viral species/strains: Bole tick virus 1, Mivirus boleense, Bole tick virus 4, Lonestar tick totivirus, Hubei toti-like virus 24 and Hulunbuir tick virus 1, as well as their genetically related variants.Phylogenetic analyses revealed substantial genetic diversity among Xinjiang tick-borne RNA viruses, ranging from highly conserved strains (e.g., Bole tick virus 3 showing >99% amino acid identity across three proteins) to novel variants (e.g., Hulunbuir tick virus 1 with 80.9% identity). Notably, Bole tick virus 1 exhibited a simplified genome architecture encoding only RdRp, suggesting potential ecological niche adaptation within Phenuiviridae, while Lonestar tick totivirus maintained the conserved GAG-POL structure characteristic of Totiviridae evolution. The cocirculation of Bole tick virus 4 and Hubei toti-like virus 24 highlights the complexity of viral ecology in ticks. These findings not only establish Xinjiang as a hotspot for TBV diversity but also underscore the imperative for further investigation into their potential zoonotic risks.

Through metagenomic NGS (mNGS) analysis, this study systematically characterized the tick-borne virome in the Urumqi region of northwestern China. Our integrated approach combining high-throughput sequencing with RT-PCR validation (confirmed by Sanger sequencing) reliably demonstrated the viral prevalence in local tick populations. The results not only expand the known genetic diversity of RNA viruses associated with *H. asiaticum* ticks but also reveal significant geographical and host-specific distribution patterns of these pathogens. Notwithstanding these findings, certain limitations warrant consideration. Notably, only *H. asiaticum* was identified in our collections, despite the known presence of other tick species in Xinjiang. This could be attributed to host specificity (as we focused on sheep and horses), ecological adaptation of *H. asiaticum* to arid environments, and seasonal activity patterns. Future studies should expand sampling to include different hosts, habitats, and seasons to better assess tick community composition in this region. Additionally, we acknowledge the importance of supplementing our study with a larger sample size and data on host infectivity to enhance the scientific rigor of our findings. The sample pooling approach, while increasing detection throughput, may have compromised sensitivity for low-titer pathogens. Furthermore, the zoonotic potential and pathogenic mechanisms of the novel viruses remain to be elucidated, requiring comprehensive investigation through cell culture models and serological assays. Building upon these results, subsequent research directions will include experimental characterization of selected TBVs' virulence and host tropism, along with expanded field surveys encompassing broader geographical coverage and larger sample sizes to establish robust epidemiological baselines for tick distributions and associated viral prevalence across the Urumqi region. Such foundational data will be critical for informing evidence-based control strategies against emerging tick-borne diseases.

## 5. Conclusion

In conclusion, our study presents the metagenomic sequencing results of pooled tick samples from various hosts in the Urumqi region of China, identifying 11 distinct RNA viruses spanning multiple families. The detected virome is characterized by a high prevalence of double-stranded RNA viruses and a substantial proportion of unclassified viruses, indicating a rich viral diversity. Through genomic and phylogenetic analyses of the six most abundant viruses, we observed significant genetic diversity and a range of evolutionary relationships. Our results also suggest that host species may affect TBVs diversity. Although our study confirmed the presence of these TBVs using RT-PCR and Sanger sequencing, we acknowledge potential limitations, including the possible omission of low-abundance pathogens. The pathogenic potential of the newly identified viruses to humans and mammals remains uncertain, underscoring the necessity for further research and broader sample collection to deepen our understanding of TBVs and to develop effective preventive measures against associated diseases.

## Figures and Tables

**Table 1:** Primer design related information.

Virus name	Primer sequences	Target fragment size (bp)
Bole tick virus 1	5′-CTCAGGACTCTTTTGCTACGC-3′ 5′-ACCTCTTTCCCTTCCCATG-3′	952

Bole tick virus 2	5′-GGAAGGACAAGAAGGAGGGT-3′ 5′-TCCAGCGGACAGAGCTAATA-3′	763

Bole tick virus 3	5′AAGCGTGACTCCATTAGCCATATTG-3′ 5′-AGCGACTCTTCAGCCTCCTTC-3′	344

Bole tick virus 4	5′-TCATTCCTCAACGCCGTCCTG-3′ 5′-TCGTGGGGCTCGCACCTAATA-3′	299

Lonestar tick totivirus	5′-GAAACCAGGCAGAAGATAACG-3′ 5′-GCTACGCTCAGAAGCAAACA-3′	264

Taishun tick virus	5′-TTCTGGGGCAACCTACAATC-3′ 5′-ATCCTCGTGGTCACTTCCTCT-3′	217

**Table 2:** RT-PCR reaction system of TBVs conserved genes.

Reagent name	Volume (µL)
2×Taq PCR MasterMix II	25
Forward primer	2
Reverse primer	2
DNA template	4
RNase free water	Up to 50

**Table 3:** TBVs conserved genes RT-PCR reaction conditions.

Procedure	Temperature (°C)	Time	Number of cycles
Predenaturation	94	3 min	1
Denaturation	94	30 s	35
Annealing	54	30 s	
Extension	72	1 min	
Final extension	72	5 min	1

**Table 4:** High-throughput sequencing data and assembly data.

Samples	Raw base (G)	Clean reads	Virus reads	Contigs total num	Max len	N50	GC (%)	Reads used (%)
H	13.3	70,457	297	92	9254	3820	58.6	80.0
S	14.0	58,350	13,672	106	9241	2732	58.1	64.3
F	18.0	2,133,455	20,962	57	7913	2722	53.9	85.7

*Note:* F, questing ticks; H, blood-feeding ticks on horse; S, blood-feeding ticks on sheep.

**Table 5:** Information on tick-borne viral sequences.

Virus name	Length (bp)	GC%	ORF	Amino acid identity (%)	Completeness	Reference virus
Bole tick virus 1/XJ/2024	6475	45.9	RdRp	99.4	Complete RdRp	Bole tick virus 1 isolate TIGMIC_1
Bole tick virus 3/XJ/2024	11,038	49.0	RdRp	99.3	Yes	Bole tick virus 3 strain BL199
			Glycoprotein	99.5		
			Nucleoprotein	99.4		
Bole tick virus 4/XJ/2024	16,234	56.4	Polyprotein	98.3	Yes	Bole tick virus 4 strain BLP-1
Lonestar tick totivirus/XJ/2024	8695	57.1	CP	96.8	Yes	Lonestar tick totivirus isolate TIGMIC_2
			RdRp	97.2		
Hulunbuir Totiv tick virus 1	5535	54.7	RdRp	80.9	No	Hulunbuir Totiv tick virus 1 isolate Hebei_037
			Hypothetical protein 1	74.4		
Hubei toti-like virus 24/XJ/2024	5797	56.6	Hypothetical protein 1 hypothetical protein 2	97.0 94.0	Yes	Hubei toti-like virus 24 strain tick 106,628

## Data Availability

The data that support the findings of this study are available from the corresponding author upon reasonable request.

## Supporting Information

Supporting Information Table S1: Primer information. Table S2: Bioinformatics software commands and parameters. Table S3: statistics of viral sequences identified by different methods. Figure S1: tick morphology. (A) Dorsal view of female tick, (B) ventral view of female tick, (C) dorsal view of male tick, (D) ventral view of male tick, (E) dorsal view of engorged female tick, and (F) ventral view of engorged female tick. Figure S2: molecular biological identification results of tick species. (A) PCR identification results of *COI* gene in tick samples and (B) phylogenetic tree of tick species based on *COI* gene. Figure S3: comparison of the number of contigs between RNA viruses and unknown viruses obtained by different softwares. Figure S4: macrovirus sequencing species classification annotations (family level).
