# Supplementary material for: Metagenomic Analysis of Tick-Borne Viruses Associated With Hyalomma asiaticum From Different Hosts in the Surrounding Areas of Urumqi, China
Source: Transbound Emerg Dis. 2025 Nov 10;2025:9985595. doi: 10.1155/tbed/9985595 (PMC12623074; doi:10.1155/tbed/9985595)
Supplement: Supporting Information — Table S1: Primer information. Table S2: Bioinformatics software commands and parameters. Table S3: statistics of viral sequences identified by different methods. Figure S1: tick morphology. (A) Dorsal view of female tick, (B) ventral view of female tick, (C) dorsal view of male tick, (D) ventral view of male tick, (E) dorsal view of engorged female tick, and (F) ventral view of engorged female tick. Figure S2: molecular biological identification results of tick species. (A) PCR identification results of COI gene in tick samples and (B) phylogenetic tree of tick species based on COI gene. Figure S3: comparison of the number of contigs between RNA viruses and unknown viruses obtained by different softwares. Figure S4: macrovirus sequencing species classification annotations (family level). [file 9985595.f1.docx]

Table S1. Primer information

| Gene | Primer sequences | Target fragment size/bp |
| --- | --- | --- |
| COI | 5′-GGTCAACAAATCATAAAGATATTGG-3′  5′-TAAACTTCAGGGTGACCAAAAAATCA-3′ | 710 |

Table S2 Bioinformatics software commands and parameters

| Software name | Commands |
| --- | --- |
| Trimmomatic | trimmomatic PE <1.fq.gz> <2.fq.gz> <1.pe.fastq> <1.unpe.fastq> <2.pe.fastq> <2.unpe.fastq> LEADING:3 TRAILING:3 SLIDINGWINDOW:5:20 MINLEN:50 -phred33 <ILLUMINACLIP:TruSeq3-PE.fa>:2:30:10 |
| BWA | bwa index -a bwtsw <refseq.fa>  bwa mem -t 12 <refseq.fa> -1 <1.clean.fq> -2 <2.clean.fq> > refseq.sam |
| MEGAHIT | megahit -t 1 -1 <1.clean.fq> -2 <2.clean.fq> --out-dir <directory> --k-min 35 –k max 95 --k-step 20 --presets meta-large --min-contig-len 500 |
| Sourmash | sourmash search <fq.gz> <final.contigs.fa> –containment |
| DeepVirFinder | python dvf.py <-i input_fa> <-o output_dir> <-l cutoff_len> <-c core_num> |
| Virsorter2 | virsorter run -w <output> -i <input> |
| geNomad | genomad end-to-end --cleanup --splits 8 <input> <output> genomad_db |
| Salmon | salmon quant –validate Mappings -i /home/salmon/ -l A -p 3 --meta -1 <1.fq.gz> -2 <2.fq.gz> |
| prodigal | prodigal -p meta -a protein_seq.fasta -m -d nucleotide_seq.fasta -o genes.gff -f gff -s poteintial.stat -i <input> |
| CD-HIT | cd-hit -i db -o db90 -c 0.9 -n 5 -M 16000 –d 0 -T 8 |

Table S3. Statistics of viral sequences identified by different methods

| Virus type | Total num | Max len | Min len | N50 | GC（%） |
| --- | --- | --- | --- | --- | --- |
| confirmed viral contigs | 34 | 5795 | 382 | 2716 | 54.20 |
| suspected viral contigs | 215 | 9253 | 319 | 4183 | 57.21 |


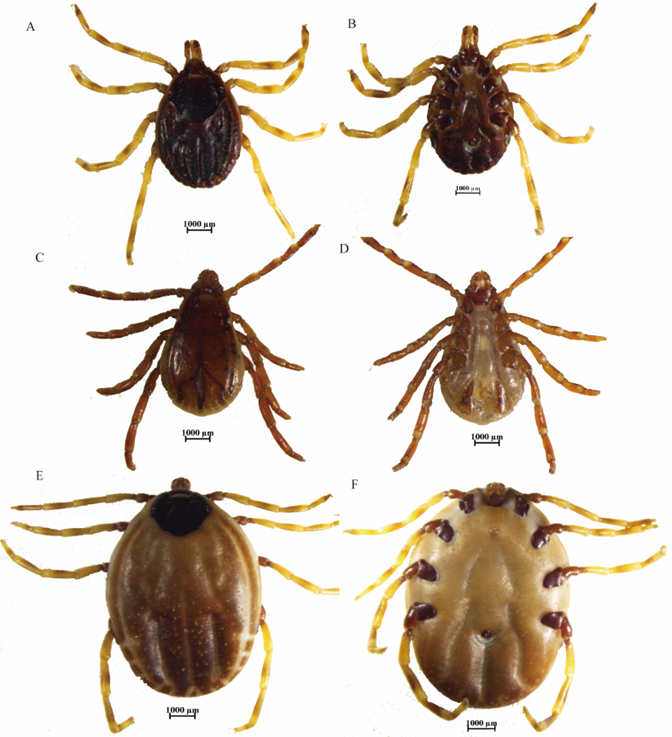


Fig. S1. Tick morphology. (A) Dorsal view of female tick, (B) Ventral view of female tick, (C) Dorsal view of male tick, (D) Ventral view of male tick, (E) Dorsal view of engorged female tick, (F) Ventral view of engorged female tick.


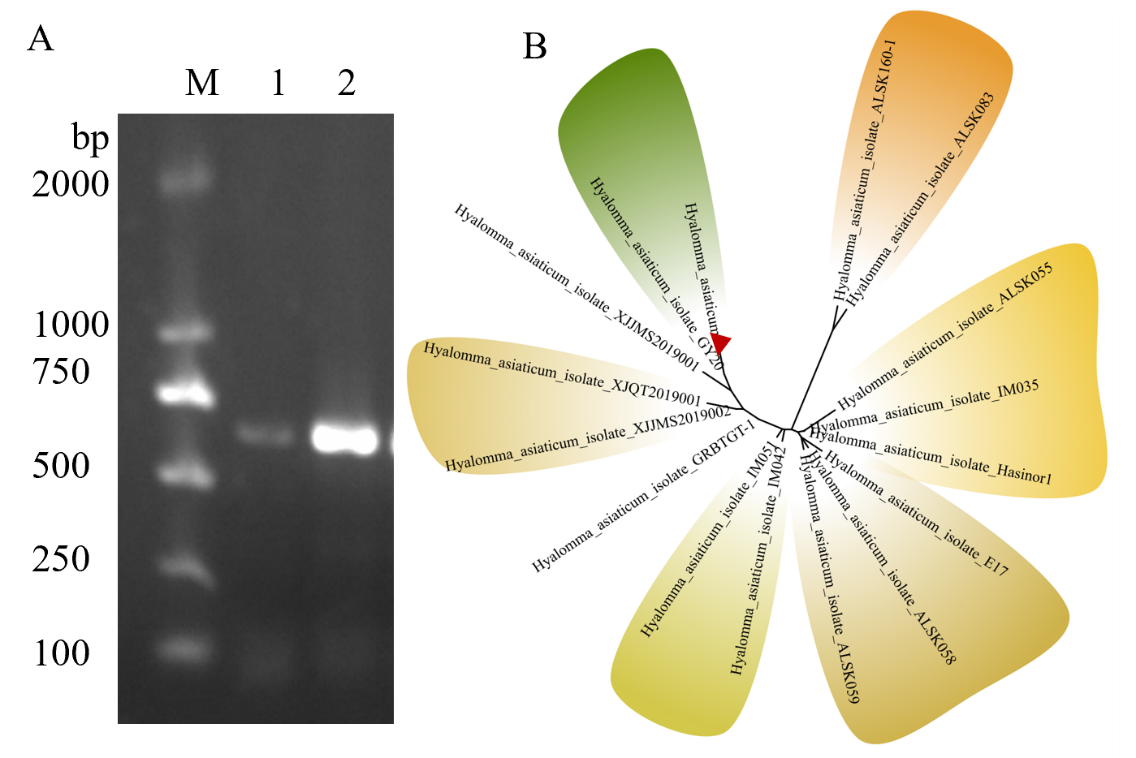


Fig. S2. Molecular biological identification results of tick species. (A) PCR identification results of COI gene in tick samples, (B) Phylogenetic tree of tick species based on COI gene.


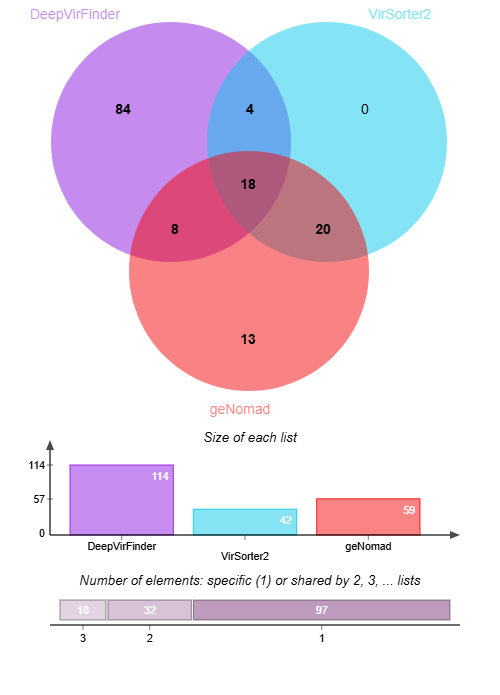


Fig. S3. Comparison of the number of contigs between RNA viruses and unknown viruses obtained by different softwares.


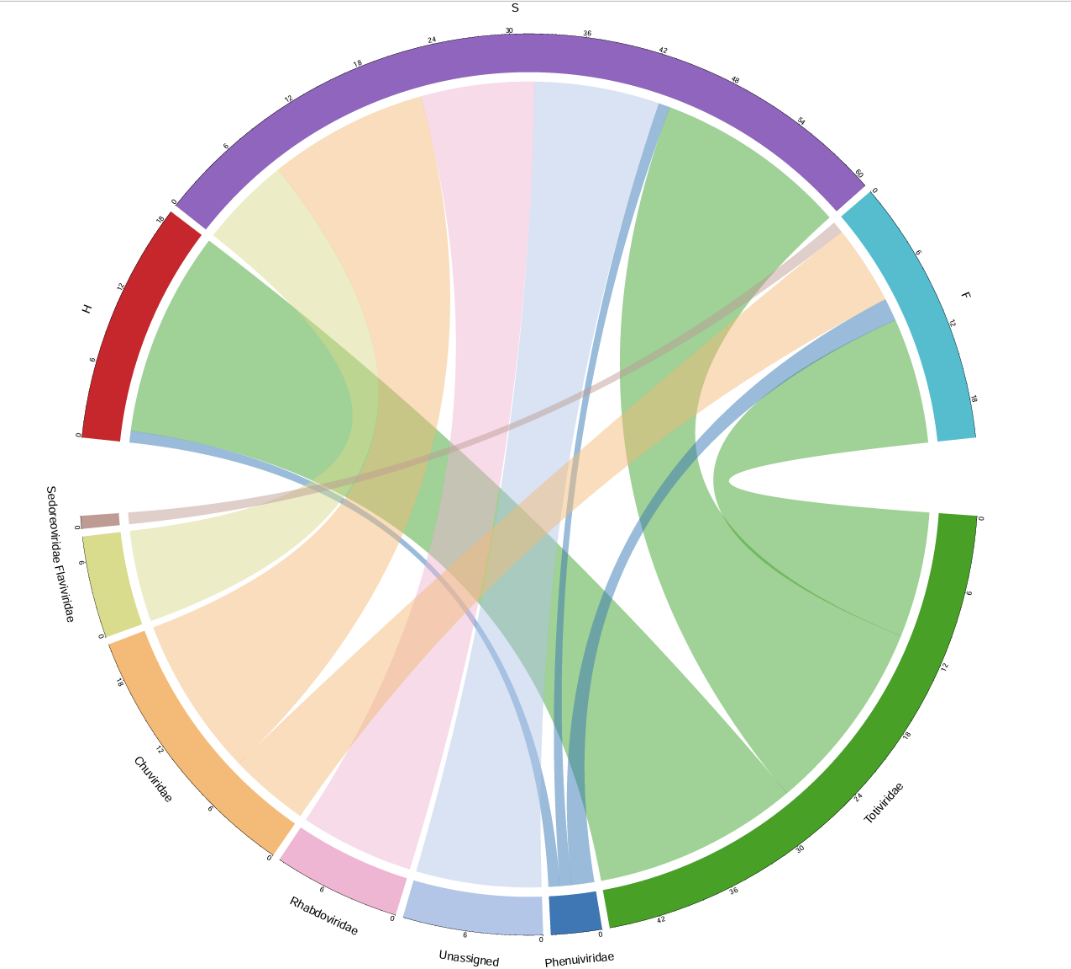


Fig. S4. Macrovirus sequencing species classification annotations (family level)
